# Stray Thoughts on Dental Hygiene

**Published:** 1901-01

**Authors:** A. F. Davenport


### STRAY THOUGHTS ON DENTAL HYGIENE.[1]

BY DR. A. F. DAVENPORT.

PERHAPS there is no subject in the realm of scientific inquiry and medical or dental research which is fraught with so much importance to the human race as a careful study of the laws of hygiene. Our organs suffer largely through our ignorance or neglect of the physiological laws which should govern them.

---

[1] Read before The New York Institute of Stomatology.

Realizing the great importance of a knowledge of the physical facts that bear upon the hygiene of the mouth, as well as upon the health of all the organs of the body, many years of undivided attention given to the care of the oral cavity leads me to speak with some confidence in regard to the dental organs.

Admitting the undeniable fact that the quality of the teeth varies according to the physiological or pathological conditions of the patient, still, were we able to commence with the young with strict hygienic habits in regard to the mouth and teeth, a very large proportion of the work of the dentist could be dispensed with.

The most important subject with us is not only how we can perform the best operations, but what can we do in the way of encouraging prevention. We should try to understand the cause as well as the remedy, and be teachers as well as practitioners, or we shall be left behind in the twentieth century march of dental progress.

There is a good deal of force in the remark said to have been made by Dr. Holmes, that "If we would improve the general health of our children we must begin two hundred years before they are born." We do not live long enough to see the effects of our teaching on the coming generations; neither are we able to tell just how much we can do in the way of controlling the nutritive development of the teeth in embryonic life.

My observation has led me to think that most dentists are inclined to accept the situation as they find it, and are content to spend most of their time and energy in trying to remedy the defects of the dental organs, instead of trying to get at the causes which underlie the entire fabric of dental science. If the physician and dentist would give more attention to prevention, and less to the cure of disease, it would be vastly better for mankind. It is a humiliating fact that in some respects, the farther we become removed from barbarism the more we degenerate physically.

Dentistry appears to be one of the outgrowths of civilization and is a striking example of the vast difference that lies between the civilized man and the barbarian.

My observation leads me to believe that our teeth do not have work enough to do to keep them in a healthy condition. The gums to be healthy need a great amount of friction, very much more than they get in ordinary mastication; this would give us fewer cases of "pyorrhœa alveolaris" to treat. It is a very common thing for me to tell my patients that the proper use of the tooth-brush would do more for them than the services of a dentist.

Dr. Whitney, one of my classmates at the dental college, and for over forty years a practising dentist in Honolulu, says the natives there rarely need the services of a dentist; the cocoanut grows in abundance in those islands, and he tells me the natives tear all the coarse husk off the cocoanut-shell with their teeth.

If the teeth were put to harder use, fewer tooth-brushes would be needed. I am satisfied that our first thought should be the care of the teeth of the young. For many years I have done all I could to have the teachers in the schools of our city add to their daily teaching "the care of the mouth and teeth" of those under their charge, especially in the primary grades, and this is now, with us, I think, pretty generally done, so that the effects are already quite manifest. The subject of the care of the teeth of children in the schools is one which I understand has been agitated somewhat in the State of New York.

When I was in London a few years ago it was my privilege to hear a report read by Dr. Cunningham, of Cambridge, from which I noted a few facts, which showed that the subject of the care of the teeth of children in the schools of England was receiving a good deal of attention. This report showed that the teeth of some two hundred thousand children had been examined. Among other places in England the teeth of the children in the schools of Hanwell were also examined, which resulted in the appointing of a dentist at a salary of eighty pounds sterling per year. The number of children at Hanwell was one thousand; of that number, nine hundred and three were carefully tabulated; each child was to have a tooth-brush and to pass through the hands of a dentist twice a year. This policy, which is being agitated in England and also in this country, is certainly one of hope for the coming generations. The kindergarten and primary teachers should bring up the subject of mouth sanitation and tooth preservation before their classes. For instance, let the teacher say, among other morning exercises, "As many as have brushed their teeth this morning, raise their hands." "All those who have not brushed their teeth this morning, raise their hands." "Also all those who have not a tooth-brush, raise their hands," etc., etc. Some such exercise as this could not fail to have a good effect.

I understand that a bill was some years since introduced into the New York Legislature to make teaching in regard to the care of the teeth of children compulsory in the public schools. I should be glad to hear what progress is being made by the "Empire State" in this direction.

A writer in the *Educational Review,* upon physical training, says, "If the school authorities will undertake this work, there is no way in which it can be so well and economically done as by making physical training a part of the school curriculum."

Formerly physical training has not included the care of the teeth, which I trust in the near future it may. When prizes are offered in our schools for the strongest muscles, the straightest back, the best chest expansion, the rosiest pair of cheeks, they should also include the best-cared-for mouth and teeth.

I have no doubt, if the mouths of the children in our schools could be examined by competent persons, carious or diseased teeth treated or removed, and instruction given and enforced with regard to the intelligent use of the brush and daily care of the mouth, the death-rate of this country would be very materially lessened, the percentage of illness much reduced, and a stronger and more vigorous race result in consequence of these prophylactic measures.

In Paris, this year, is to be held an athletic festival, and it is hoped that the great progress that is being made in physical culture throughout the world will also include the organs of mastication.

When a patient takes my chair I always make it a point to first note the condition of the mouth, and make any suggestions I think necessary as to its care, giving them to understand that the care they daily give their mouth and teeth is of far greater importance than any service I can render; in other words, I do all I can to make my patients self-supporting.

I have not chosen this subject thinking it was in any respect a new one; in fact, I am well aware that it would be presumption for me to attempt to bring anything new in dentistry to such an intelligent body of practitioners as I see before me, fully realizing that your field furnishes far greater opportunities for observation than does a practice in a small country city. I have chosen this subject more especially because it is one that worries me most, in hopes that by so doing I might hear something from you that will help me to solve the perplexing problem, as also to give you an idea of what has been the summing up of my thought after forty years of practice.

